# Facile synthesis of 2-hydroxyacetophenone from racemic styrene oxide catalyzed by engineered enzymes

**DOI:** 10.1007/s10529-022-03271-w

**Published:** 2022-06-22

**Authors:** Isac Söderlund, Elias Tjärnhage, Emil Hamnevik, Mikael Widersten

**Affiliations:** Department of Chemistry – BMC, Uppsala University, Box 576, 751 23 Uppsala, Sweden

**Keywords:** Alcohol dehydrogenase, Epoxide hydrolase, Biocatalysis, Cascade reactions, α-Hydroxy ketone

## Abstract

We describe a system that allows for biocatalyzed in vivo synthesis of α-hydroxy ketones from racemic epoxide starting material by in vivo co-expression of native and engineered epoxide hydrolase and alcohol dehydrogenases. The constructed expression system exploits the host cell metabolism for supply and regeneration of precious nicotinamide dinucleotide coenzyme. Racemic styrene oxide added to growth medium passively enters the cells and is hydrolyzed into (1*R*)-phenylethane-1,2-diol, which is subsequently oxidized to the acyloin 2-hydroxyacetophenone. Produced 2-hydroxyacetophenone escapes the cells via passive diffusion into the growth medium. Thus, co-expression of potato epoxide hydrolase and engineered alcohol dehydrogenase variants can be employed for robust and facile production of 2-hydroxyacetophenone from racemic styrene oxide.

**Supplementary Information:**

The online version contains supplementary material available at 10.1007/s10529-022-03271-w.

## Introduction

The employment of enzymes as biocatalysts have emerged as powerful and often more sustainable alternatives to traditional routes in synthetic chemistry of needed compounds (c.f. Turner and Truppo 2013). Important chemical transformations comprise oxidation/reduction of *sec*-alcohols and their corresponding ketones. Vicinal diols and the corresponding α-hydroxy ketones (acyloins) are attractive as chiral auxiliaries, ligands, templates for asymmetric reactions and as building blocks of fine chemicals and pharmaceuticals (Bhowmick and Kartick 2006; Hoyos et al. 2010; Palomo et al. 2012). Production of acyloins using biocatalysis has been presented using monooxygenase catalyzed hydroxylation of ketones (Agudo et al. 2015) or applying alcohol dehydrogenases either regioselectively oxidizing or reducing vicinal diols or diketones, respectively (Zhang et al. 2013; Maurer et al. 2018). We describe here a system for in vivo production of an acyloin that can be readily extracted from the growth medium of a host cell culture.

**Fig. 1:**
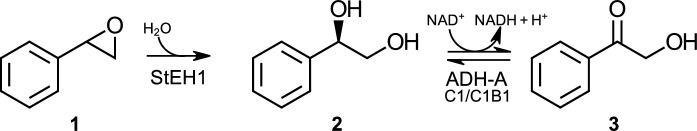
Compounds and reactions studied in the current work. Racemic styrene oxide (**1**) is hydrolyzed in a enantioconvergent reaction into (1*R*)-phenylethane-1,2-diol (**2**) by epoxide hydrolase StEH1. The resulting vicinal diol is subsequently oxidized in a regioselective transformation by engineered alcohol dehydrogenase ADH-A into the final 2-hydroxyacetophenone product (**3**)

Potato epoxide hydrolase StEH1 catalyzes the hydrolysis of styrene oxide (**1** in Fig. 1) into (1*R*)-phenylethane-1,2-diol (**2**) in an enantioconvergent reaction (Monterde et al. 2004; Janfalk Carlsson et al. 2012) resulting in an almost enantiopure diol product from racemic epoxide. This enzyme is unusually thermostable for a mesophilic protein and can withstand high concentrations of alternative water miscible solvents such as deep eutectic solvents (Lindberg et al. 2010a). The alcohol dehydrogenase A (ADH-A) from the bacterium *Rhodococcus ruber* DSM 44541 is an interesting enzyme candidate for biocatalytic redox transformations. It exhibits unusual tolerance towards water miscible organic solvents, is highly regio- and enantioselective and displays NAD^+^/NADH dependent activity with a wide range of aryl substituted *sec*-alcohols and ketones (Stampfer et al. 2002, 2003, 2004; Kosjek et al. 2004; Hamnevik et al. 2014). The activity with 1,2-diols is, however, comparably poor (Hamnevik et al. 2014) which renders this enzyme less suitable for biocatalytic acyloin synthesis. With the aim to improve on the activity with vicinal diols such as **2**, we subjected ADH-A to laboratory evolution, selecting for enzyme variants with improved activity with this diol (Hamnevik et al. 2018). Two enzyme variants were isolated (F43H, dubbed ‘C1’ and F43H/Y54L, ‘C1B1’) that both displayed improved turnover numbers in the catalyzed regioselective oxidation of **2** into 2-hydroxyacetophenone (**3**). The goal with the current work was to investigate the possibility for synthesis of **3** from racemic** 1** by exploiting the stereoselectivities of the described enzymes in series. We aimed for in vivo expression in *Escherichia coli* of the enzymes and of the catalyzed reactions, thereby taking advantage of the host.

cell’s metabolism for nicotinamide coenzyme supply and recycling.

## Materials and methods

### Chemicals, reagents and software

All chemicals, reagents, microbial growth media and protein purification resins were purchased from commercial sources and at highest available quality. Oligonucleotides (Table S1 in the Supplementary Information) were supplied by Thermo Fisher Scientific and the pETDuet-1 vector was supplied by Novagen. Endonucleases were supplied by Thermo Scientific. GeneJET Gel Extraction Kits and GeneJET Plasmid Miniprep Kits from Thermo Scientific were used extraction of fragment and plasmid DNA. Chelating Sepharose Fast Flow gel supplied form GE Healthcare was used for the immobilized metal ion affinity chromatography (IMAC). Either a NanoDrop® ND-1000 Spectrophotometer or a UV-1700PharmaSpec UV–VIS spectrophotometer from Shimadzu was used for spectrophotometric measurements.

### Gene/cDNA cloning

The respective genes (ADH-A F43H and F43H/Y54L) or cDNA (StEH1) were amplified by PCR and subcloned into pETDuet-1 as described in Fig. S1. The resulting expression plasmids were named pETDuetADHC1StEH1 or pETDuetADHC1B1StEH1, respectively (Fig. S2).

### Enzyme expression and purification

Enzymes were expressed in *E. coli* strain BL21-AI (Novagen) and purified as described before using Ni(II) IMAC (Elfström and Widersten 2005; Hamnevik et al. 2014) with the modification that expression of ADH-A variants and co-expression of chaperonins GroEL/ES were induced by addition of 0.04% (w/v) L-arabinose and 1 mM isopropyl β-D-thiogalactosidase (IPTG), respectively.

### Growth and reaction conditions

*E. coli* BL-21AI [pREP4] encoding chaperonins GroEL/ES (Dale et al. 1994) were transformed with petDuetADHC1StEH1 or petDuetADHC1B1, respectively and plated on LB (10 g/l tryptone, 5 g/l yeast extract and 10 g/l NaCl) plates containing 100 µg/ml ampicillin, 30 µg/ml kanamycin and incubated overnight at 37 °C. One colony of transformant containing either expression construct was inoculated into 2.5 ml of 2TY (16 g/l tryptone, 10 g/l yeast extract and 5 g/l NaCl) containing 100 µg/ml ampicillin and 50 µg/ml kanamycin and incubated at 25 °C, 200 rpm for 5 h. One ml of culture was transferred to 35 ml of 2TY with antibiotics as above and cultures were incubated overnight at 25 °C, 200 rpm. Three replicates of each clone were inoculated at 5 min intervals by addition of 10 ml of over-night culture to 500 ml of 2TY with 100 µg/ml ampicillin and 50 µg/ml kanamycin and culture density was monitored by measuring OD_600_ at t = 0, and followed continuously. After 4 h incubation, at OD_600_ ≈ 0.4, protein expression was induced by addition of 1.0 mM IPTG and 0.2% (w/v) L-arabinose. One hour following induction of protein expression, neat racemic styrene oxide was added to the cell culture to a final concentration of 10 mM. Five-ml aliquots were removed every hour during the first 6 h, and every second hour the following day and every third hour during days three and four of incubation. All samples were immediately cooled on ice. Samples were centrifuged at 4,000×*g*, 4 °C for 5 min. After centrifugation supernatants were transferred into a 15 ml conical tube and supernatants and cell pellets were stored at − 80 °C until analyzed.

Supernatants were thawed on ice and vortexed briefly before 1 ml was transferred to microcentrifuge tubes. Samples were centrifuged for 5 min at 17,000×*g* and filtered through a 0.45 µm PVDF membrane filter into new microcentrifuge tubes. Filtered samples were stored at − 20 °C. Pelleted samples were thawed on ice and resuspended in 500 µl water and transferred to microcentrifuge tubes. Cells were lysed in 500 µl Lysis Buffer (0.2 M NaOH, 1% (w/v) sodium dodecyl sulfate). Following mixing by inverting the tubes and incubation for 3 min, 600 µl of Neutralization Buffer (3 M potassium acetate, 11.5% (v/v) acetic acid) were added to the solutions and tubes were mixed by inverting 6 times. Tubes were centrifuged at 17,000×*g* for 3 min. The supernatants were transferred to new microcentrifuge tubes and centrifuged again for 5 min at 17,000×*g*. Samples were subsequently filtered through 0.45 µM PVDF into new tubes as described above and stored at − 20 °C awaiting analysis.

Samples were analyzed by reversed phase HPLC over an Ascentis C-18, (25 × 0.46 cm, 5 μm bead size) column using Shimadzu Providence system equipped with a LC-20AD pump. Solvent A, 50 mM sodium-phosphate, pH 3.0, solvent B, methanol applying a gradient of 30% B for 5 min with an increase to 80% B over 15 min, followed by 5 min at 80% B, and a decrase to 30% B over 5 min, and finally 30% B for 5 min. The flow-rate was 0.5 ml/min and 10 µl of filtered sample were injected. Epoxide **1** and diol** 2** diol were detected at 220 nm and ketone** 3** was detected at 244 nm using a diode array SPD-M20A. Peak identities and quantification of analytes were established from parallel runs of pure reference compounds.

## Results and discussion

Co-expression of epoxide hydrolase and alcohol dehydrogenase can provide a possible biocatalytic route to stepwise conversion of racemic styrene oxide (**1**) into the acyloin **3** (Fig. 1). An intrinsic issue when utilizing NAD(P) dependent oxidoreductase enzymes is the necessity of efficient coenzyme recycling since inclusion of stoichiometric amounts are far too costly for synthetic applications. Nonphysiologically high concentrations of NAD^+^/NADH also generally lead to severe competitive inhibition of the alcohol dehydrogenase catalyzed reaction. The system tested here was designed for intracellular enzyme expression and reactions which would allow for hijacking the cellular metabolism for supply and regeneration of the required NAD^+^ coenzyme. A related system utilizing (*S*)-epoxides as starting reactants and targeting oxidation of the primary alcohol for production of 1,2-amino alcohols has been reported which further elegantly illustrates the potential of the in vivo approach for serial reactions dependent on coenzyme recycling (Liu et al. 2019).

All expressed enzymes could be detected after expression and purification by affinity chromatography (Fig. S3). Expression levels, as judged from protein samples separated by SDS-PAGE and stained with Coomassie Brilliant Blue R-250, appear to be relatively similar between the proteins. However, following purification, the majority of dehydrogenase enzymes are lost due to apparent lower solubility and entrapment in the non-soluble (‘pellet’) fraction (Fig. S3). If the alcohol dehydrogenases are indeed aggregating after expression or an artifact of the applied lysis protocol is unknown. The amounts of soluble enzyme was at this point considered adequate.

**Fig. 2:**
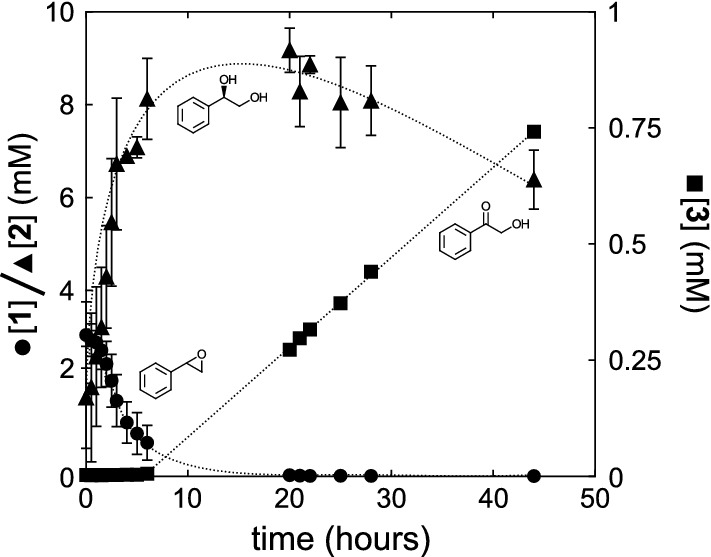
Progression of reactions leading to the final ketone product (**3**). Styrene oxide (black circle, 10 mM) was added to the *E. coli* growth medium one hour following induction of protein expression. Aliquots of the growth medium were removed for analysis of reactant, intermediate and acyloin product (black square) at different time points. The hydrolysis into diol **2** (black triangle) was rapid and the epoxide was undetectable in the growth medium after 20 h. Error bars represent standard deviation (n = 3). Dotted lines are manually inserted trend lines

The two expression *E. coli* [pETDuetADHSTEH1] constructs were tested for in vivo synthesis of **3**. Styrene oxide (**1**, 10 mM) was added to log phase bacterial cultures after one hour of induction of enzyme expression and the relative concentrations of **1**–**3** in the growth medium were analyzed at different time points (Fig. S4). Figure 2 summarizes the outcome of the reaction with StEH1 and ADH-A F43H (C1). The corresponding results when combining the ADH-A C1B1 (F53H/Y54L) variant and StEH1 were fully comparable.

StEH1 displays high activity with (*S*)-**1**, *k*_cat_/*K*_M_ = 68 s^−1^ mM^−1^ as compared to 0.99 s^−1^ mM^−1^ with (*R*)-**1** (Lindberg et al. 2010b). Thus, it is expected that the (*S*)-enantiomer is consumed at a considerably faster rate as compared to (*R*)-**1**, and the rapid decrease in the concentration of *rac*-**1** to approximately 50% may reflect that fact. The slower phase, up to 20 h, may be the hydrolysis of the less favored (*R*)-**1**. A concomitant rise in diol** 2** concentration that leveled out at approximately 8 mM was observed. Thus, only 80% of diol + epoxide was observed in the growth medium which could be due to that all epoxide, diol and ketone product did not fully partition into the growth medium. The diol is considerably more polar (ACD/logP = 0.04) as compared to the epoxide (ACD/logP = 1.61). Hence, it is expected that the rate of equilibration over the cell membrane will be slower and a portion of **2** once formed, may be trapped inside the cells. This was confirmed after analysis of reaction components concentrations in the cell fraction which contained an (normalized) estimated steady state concentration of 2 mM of the diol intermediate resulting in a steady-state ratio of **2**^outside^/**2**^inside^ of approximately 3.5.

The more sluggish oxidation activities of the ADH-A variants, *k*_cat_/*K*_M_ = 0.050 s^−1^ mM^−1^ for both variants (Hamnevik et al. 2018), delayed the formation of ketone **3** which increased linearly in concentration over two days to approximately 0.8 mM. The main reason for the lag phase preceding detection of the ketone product in the growth medium is most probably due to the high values of *K*_M_ for diol **2** that both ADH-A variants display, 37 and 120 mM for C1 and C1B1, respectively. Thus, the reaction velocity of the oxidation reaction will become very low until appreciable concentrations of the diol intermediate have accumulated. The relatively low yield (~ 10%) is in accordance with the thermodynamic equilibrium which lies strongly towards the diol state. The observed ratio of diol/ketone at the presumed equilibrium would correspond to a Δ*G* of 5.5 kJ/mol, a value lower and far more favorable in the direction of ketone formation as compared to standard free energy changes of many comparable NAD^+^ dependent alcohol oxidation reactions. The fact that the ketone product is expected to escape the cells (ACD/logP = 0.44) more efficiently as compared to diol **2**, can shift the equilibrium towards a relatively larger proportion of the end product. Analysis of normalized amounts of **3** in the cell fraction supported this notion; the ratio of **3**^outside^/**3**^inside^ was estimated to approximately 25 after 70 h.

The concentrations of reactants and products were in this study far from relevant production scales but the results demonstrate promising starting points for optimization and up-scaling as well as refinement of downstream product isolation. The value increase from starting material to product, estimated from published list prizes of common manufacturers, corresponds to 50 to 100-fold. Further engineering on behalf of the alcohol oxidation reaction is expected to shorten the reaction time. Yields are mainly limited by the thermodynamic barrier, yet favored by the fact that the product ketone escapes the cells more efficiently as compared to the intermediate diol.

## Supplementary Information

Below is the link to the electronic supplementary material.

Supplementary file1 (PDF 494 kb)
