# Supplementary material for: Facile synthesis of 2-hydroxyacetophenone from racemic styrene oxide catalyzed by engineered enzymes
Source: Biotechnol Lett. 2022 Jun 22;44(8):985–90. doi: 10.1007/s10529-022-03271-w (PMC9356933; doi:10.1007/s10529-022-03271-w)
Supplement: Supplementary file 1 — Supplementary file1 (PDF 494 kb) [file 10529_2022_3271_MOESM1_ESM.pdf]

**Supplementary Information**

# Facile Synthesis of 2-Hydroxyacetophenone from Racemic Styrene Oxide Catalyzed by Engineered Enzymes

Isac Söderlund, Elias Tjärnhage, Emil Hamnevik, and Mikael Widersten*

Department of Chemistry – BMC, Uppsala University, Box 576, SE-751 23 Uppsala, Sweden

*Corresponding author: E-mail: mikael.widersten@kemi.uu.se, ORCID: 0000-0002-3203-3793

INDEX

**Supplementary table**……………………………………………………………….………….... **S2**

**Table S1.** Oligonucleotides used………………………………………………………………. S2

**Supplementary figures**……………………….…………………………………………….……… **S2**

**Fig. S1.** Schematic description of subcloning strategy……………………………….………….. S2

**Fig. S2.** Plasmid construct used in enzyme co-expression…………………….…………………. S3

**Fig. S3.** SDS-PAGE analysis of protein expression……………………………………………… S3

**Fig. S4.** Sample C-18 HPLC chromatograms……………………………………….…………… S4

**Table S1.** Oligonucleotides used

| Name | Sequence 5'→ 3' |
|---|---|
| ADH-A forward | TTT TTT CCA TGG ATG AAA GCC GTG CAG TAT ACC |
| ADH-A reverse | TTT TTT AAG CTT TCA TTA ATG ATG ATG ATG ATG ATG CGG AAC AAC AAC ACC GCG ACC |
| StEH1 forward | TTT TTT CTG CAG CAT ATG ATG AAG AAG ATA GAG CAC AAG ATG |
| StEH1 reverse | TTT TTT CTC GAG TCA TTA ATG ATG ATG ATG ATG ATG AAA CTT TTG AAT GAA GTC ATA GAT |

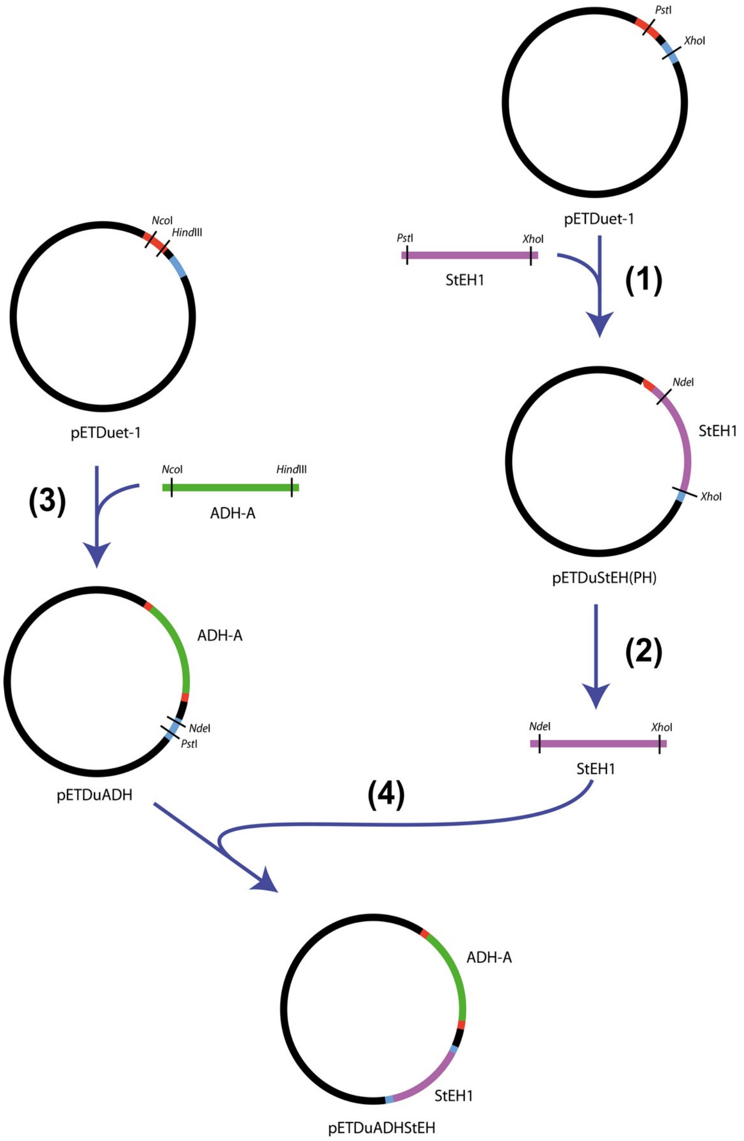


**Fig. S1.** Schematic description of subcloning strategy of StEH1 cDNA and genes for either ADH-A F43H or F43H/Y54L: (1) The PCR product encoding StEH1 was inserted into the *Pst*I/*Xho*I sites of pETDuet-1. (2) The cDNA fragment was subsequently removed from the plasmid using *Nde*I and *Xho*I, and purified. (3) The PCR fragments encoding ADH-A Y43H or F43H/Y54L were subcloned in parallel into the *Nco*I/*Hin*dIII sites of pETDuet-1. (4) Finally, the StEH1 cDNA was inserted into the *Nde*I/*Xho*I sites of the two different pETDuADH derivatives to generate the final construct containing the ADH-A gene and StEH1 cDNA (Fig. S2).

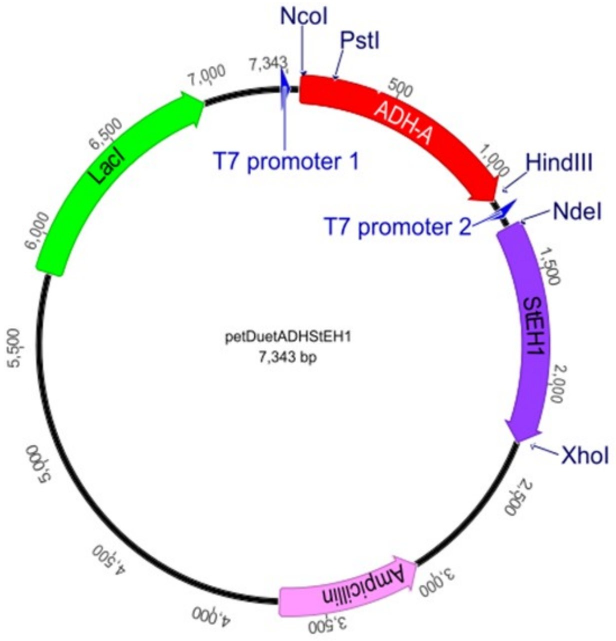


**Fig. S2.** Plasmid construct used in the co-expression of alcohol dehydrogenase variants and epoxide hydrolase. Restriction enzymes *Nco*I, *Hin*dIII, *Nde*I and *Xho*I were utilized as described in Fig. S1.

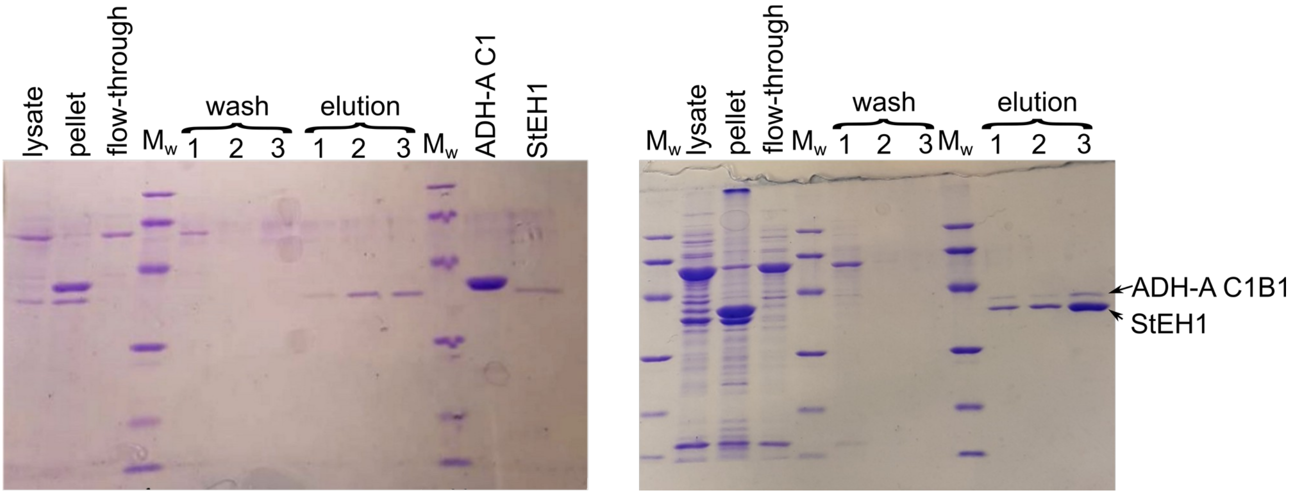


**Fig. S3.** SDS-PAGE analysis of protein expression and Ni(II)-IMAC purification. Left panel: Samples from different stages of purification following co-expression of StEH1 and ADH-A F43H ('C1'). Right panel: Samples from different stages of purification following co-expression of StEH1 and ADH-A F543H/Y54L ('C1B1'). It is clear that the majority of produced ADH-A enzyme is trapped in the insoluble 'pellet' fraction in both cases.

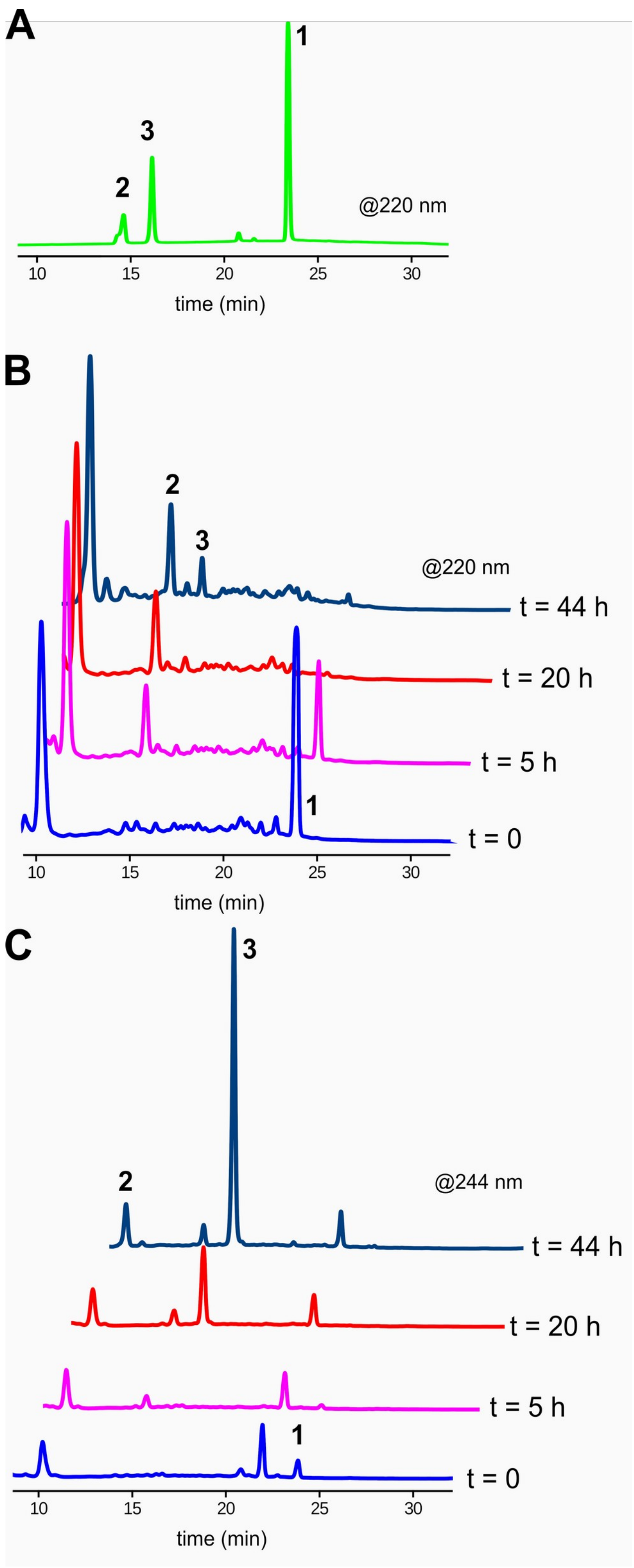


**Fig. S4.** Sample C-18 HPLC chromatograms of crude samples of growth medium removed from the culture at given time points. See main text for details of sample preparation and solvent gradient. (**A**) Separation of reference compounds. 2 mM of styrene oxide (**1**), phenylethane-1,2-diol (**2**) and 2-hydroxuacetophenone (**3**) detected at 220 nm. (**B**) and (**C**) Samples of growth medium analyzed after the given incubation times after addition of 10 mM styrene oxide, detected at (**B**) 220 nm, or (**C**) 244 nm.
